# Building a culture of engagement at a research centre for childhood disability

**DOI:** 10.1186/s40900-021-00319-5

**Published:** 2021-11-06

**Authors:** Kinga Pozniak, Francine Buchanan, Andrea Cross, Jennifer Crowson, Barb Galuppi, Danijela Grahovac, Jan Willem Gorter, Oksana Hlyva, Marjolijn Ketelaar, Olaf Kraus de Camargo, Manda Krpan Mesic, Rachel Martens, Dayle McCauley, Linda Nguyen, Robert J. Palisano, Michelle Phoenix, Connie Putterman, Peter Rosenbaum, Jennifer Sprung, Sonya Strohm, Rachel Teplicky, Donna Thomson, Marilyn Wright

**Affiliations:** 1 CanChild Centre for Childhood Disability Research, McMaster University, Hamilton, Canada; 2 Department of Pediatrics, McMaster University, Hamilton, Canada; 3 School of Rehabilitation Science, McMaster University, Hamilton, Canada; 4 Down Syndrome Association of Hamilton, Hamilton, Canada; 5 Center of Excellence for Rehabilitation Medicine Utrecht, UMC Utrecht Brain Center, University Medical Center Utrecht, and De Hoogstraat Rehabilitation, Utrecht, The Netherlands; 6 Drexel University, Philadelphia, PA USA; 7 Holland Bloorview Kids Rehabilitation Centre, Toronto, Canada

**Keywords:** Family engagement in research, Childhood disability, Organizational ethnography, Collaborative auto-ethnography

## Abstract

**Background:**

Engaging patients and family members as partners in research studies has become a widespread practice in healthcare. However, relatively little has been documented about what happens after the research study ends. For example, is patient and family engagement embedded in the wider infrastructure of organizations, and if so how? What are the long-term effects of engaging parents on research teams on the culture of how research is conducted? This study seeks to address these two gaps by examining how a culture of family engagement has been built over time at CanChild Centre for Childhood Disability Research at McMaster University in Ontario, Canada.

**Methods:**

This study is based on ethnographic research methodology and combines elements of organizational ethnography, interviews, and collaborative auto-ethnography with parent partners, researchers, staff, and trainees.

**Results:**

Since the inception of CanChild Centre for Childhood Disability Research at McMaster University in 1989, parents have been involved in research studies. Over time, this involvement evolved from being consulted on research studies to undertaking decision-making roles as partners and most recently as co-principal investigators. A growing infrastructure fosters a community of engagement that goes beyond the individual research study, and often beyond CanChild. This infrastructure consists of training, knowledge mobilization and social networking. In addition, the “softer” building blocks of CanChild’s culture of engagement are an openness to learning from others, a commitment to relationship building, and a drive to grow and improve. These values are espoused by the leadership and are instilled in the next generation of researchers to inform both research and clinical work. While some challenges should be acknowledged when researchers and family partners work together on research studies, we identify a number of strategies that we have used in our studies to foster authentic and meaningful family–researcher partnerships.

**Conclusion:**

Engaging patients and families as partners in research constitutes a culture shift in health research, whereby studies about patients and families are carried out with them. Developing a community of engagement that transcends an individual research study is a step towards creating a culture of research that is truly shaped by the people about whom the research is being done.

## Plain English summary

More and more patients and family members are getting involved in health research studies as partners. However we do not know much about what happens after the research study ends. This article looks at how parents have been involved in research studies at CanChild Centre for Childhood Disability Research at McMaster University in Ontario, Canada. CanChild researchers, staff, students and parents were asked about their experiences of working together on research studies. One of the researchers then pooled together all of these stories, shared them with everyone to get their feedback, and wrote the initial draft of this article. All the people interviewed were invited to read the article and to add their thoughts and opinions until everyone was satisfied with the final product. Our shared stories show that a lot has changed since CanChild was established in 1989. At first, researchers consulted with parents when they were doing a study. Now, many parents are partners and co-principal investigators on research studies. CanChild has also developed opportunities for parents and researchers to get training in patient-family engagement and to network with each other outside of research studies. Researchers, staff, students and parents talked about what makes research partnerships successful, including: being open to learning from each other; taking the time to get to know each other as people; and always trying to do better. They also shared some of the challenges that come up on research studies and suggested strategies for working through them.

## Background

Engaging patients and family members as partners in research studies is a growing trend, and often an expectation, in healthcare. This practice has spurred the development of new engagement frameworks, “how-to” guides, syntheses and reflections on lessons learned during research studies [1–5]. It has also given rise to efforts to measure and evaluate the impact of patient and family engagement (also known as patient and public involvement) on the patients and caregivers themselves, the research projects in which they partake, and the research community as a whole [6–13]. However, to date relatively little information has been documented about patient and family engagement beyond the individual research study: for example, how it is embedded in the wider infrastructure of organizations [14–16] or what the long-term effects are on programs of research, institutions, and ultimately the culture of how research is conducted [4].

This study seeks to address these two gaps in the literature by examining the evolution, building blocks and infrastructure of family engagement at a research centre for childhood disability. The CanChild Centre for Childhood Disability Research was founded in 1989 and is based at McMaster University in Hamilton, Canada. It is an academic network of local, national and international scientists who conduct applied clinical and health services research in the field of childhood disability and rehabilitation. For three decades, CanChild researchers have collaborated with members of various groups (including families, youth, service providers and policymakers) in research studies and other initiatives. In this article, the authors—composed of CanChild scientists, staff, trainees and parent affiliates—describe and analyze CanChild’s trajectory of working with parents of children with disabilities.

This study was envisioned by KP, a postdoctoral researcher at CanChild, and supervised by JWG, a clinician-scientist and the director of CanChild. JWG has twenty years of experience leading research studies that involve parents in various roles and capacities. Under his leadership, a knowledge translation strategic plan was developed with engagement of families in childhood disability research as a priority for CanChild [20]. KP is a socio-cultural anthropologist and a mother to a child with cerebral palsy. In her role at CanChild, she contributes to several research studies that engage parents in different ways, including a training program for families and researchers in Family Engagement in Research (FER). In her dual role as a researcher and parent partner, she contributes to family advisory committees for two research studies. She is also a member of CanChild’s private parents’ Facebook group called ‘Parents Partnering in Research’. Through partaking in these activities, she has developed a research interest in parents’ participation in research. One of the questions pursued in her study is how parent engagement at CanChild has evolved and how it is being cultivated.

This study reveals that parents’ involvement at CanChild evolved from them being consulted on research studies to undertaking decision-making roles as partners and sometimes co-principal investigators. The building blocks and facilitators of parent–researcher partnerships include an organizational infrastructure of training, knowledge mobilization and social networking, underpinned by the values of openness to learning from others, a commitment to relationship building, and a drive to grow and improve. We outline the key issues we have encountered while working together on research studies, and suggest strategies for addressing them. Throughout, we aim to situate the individual experiences of researchers and parents and the organizational practices of CanChild in the context of larger trends in health research.

## Methods

This study is grounded in ethnographic research methodology and combines elements of organizational ethnography, interviews, and collaborative auto-ethnography. Originating in the anthropological tradition, ethnography is best understood as a methodology rather than merely a method of collecting data. Anthropology has traditionally been defined as the study of people and cultures, and ethnography has been its flagship methodology used to study cultural processes. Ethnographers seek to understand and describe what people do, what they say about what they are doing, what it means to them, and what it signifies about larger social, cultural, political and economic patterns, processes and phenomena [17]. The classic method associated with ethnographic research is participant observation. Broadly speaking, this consists of being embedded in the everyday lives of people whom one is researching, and, as much as possible and appropriate, doing whatever it is that they are doing [18, 19]. Participant observation is often used in conjunction with other methods, such as interviews. The knowledge produced through the interactions between a researcher and the people with whom they are speaking is considered intersubjective or co-produced. For this reason, ethnographers are reflective about their own identities and social locations, and how these shape the entire research process.

Given our research focus on the evolving culture of parent engagement, we have deemed ethnography to be a fitting methodology for this study. In particular we have chosen an approach called organizational ethnography, which refers to doing ethnography in, and of, organizations. This approach recognizes that organizations have their own “affiliations, identities, histories, and cultures, some of which are properties of the organization as a whole” and that what happens in an organization is mediated by its “mission, culture, history, policies, and structure” [21]. This perspective allows us to treat CanChild both as a site where parent engagement is being done, and as a unit of analysis in itself. In this article we explore the extent to which being its own entity housed within a university has enabled CanChild to develop a particular organizational dynamic and infrastructure of patient and family engagement.

JWG invited all interested CanChild researchers, trainees, staff and parent affiliates to participate in an interview with KP to explore their experiences with parent engagement. The study was approved by the Hamilton Integrated Research Ethics Board (HiREB) and all participants provided written consent. Twenty-three individuals in total took part in an interview: six researchers/clinicians, five staff, two research trainees and ten parents. The interviews were semi-structured and designed to capture participants’ experiences with taking part in research studies or other initiatives in which researchers and parents worked together. KP asked researchers, staff and trainees to describe situations in which they engaged parents as “more than participants” in a research study, their rationale for doing so, how they found working with parents, what they learned, and what challenges they encountered. Parents were asked to describe the research studies in which they have been engaged as “more than participants”; what motivated them to get involved; what it was like for them to partner on a research study; what benefits or learnings they got out of it; and what challenges they encountered. Both researchers and parents were invited to reflect broadly on the role of parent engagement in childhood disability research, its contributions and challenges, and how they see it unfolding in the future. Some of the interviewees provided specific examples from various research studies in which they have been involved (whether at CanChild or elsewhere), while others contributed more meta-level reflections about trends and issues in the field. As the majority of parents had experiences with research studies outside of CanChild, they were able to comment broadly on issues surrounding parent engagement, to identify changing trends in the Canadian landscape over the past decade, and to suggest areas that need to be addressed in the future.

The interviews were recorded and transcribed by KP and each participant was offered the opportunity to review, edit, or redact their own transcript. The transcripts were analysed and coded by KP using thematic analysis, with themes derived both inductively and deductively, driven by the interview questions as well as by the existing literature on patient engagement [22]. Data analysis proceeded iteratively, with KP revisiting and reorganizing the thematic frame. KP then shared a list of emerging themes with the interviewees to give further opportunities for comment. KP also presented preliminary findings at CanChild’s research rounds, which was followed up by another invitation for anyone else who was interested to contribute to the project.

Throughout this process, KP realized that participants’ individual accounts reflected a collective CanChild voice: many participants addressed the same issues, and explicitly connected their individual experiences to what they perceived were broader cultural values and structures at CanChild. At that point KP decided to expand the methods and capture this collective meaning-making through an approach called collaborative auto-ethnography. Though all ethnography is inherently reflexive, auto-ethnography explicitly uses the researchers’ own experiences and reflections as data. Researchers work together to collect, analyze and interpret their data in order to gain a meaningful understanding of the phenomena reflected in their autobiographical data [23, 24]. The process is iterative, since individual meaning-making takes place alongside group meaning-making, which is negotiated among participants.

KP invited all interviewees to contribute to this article as co-authors and all except two accepted. KP wrote the first draft of the article and circulated it to all co-authors for feedback. Due to the confidential nature of interviews, the authors did not read each others’ interview transcripts; rather, they commented on the draft summarizing de-identified results. The authors had the option to review a Google document to enable them to see and respond to each others’ comments, or review the draft on Microsoft Word and provide feedback independently. The draft was revised and recirculated until a consensus was reached on the final version. This process allowed all the co-authors to reflect on, and interpret, both their own experiences and the experiences shared by others. In effect, the ensuing depiction of CanChild presented below is not merely KP’s own interpretation of the experiences related to her by participants, but rather the product of collective meaning-making on the part of twenty-three co-authors.

## Results/discussion

We describe the ways in which parents’ roles in research have evolved at CanChild from (1) parents contributing in consultative roles to undertaking decision-making roles; (2) parents’ involvement in individual research studies to ongoing involvement in the general research program at CanChild; and (3) siloed incidences of engagement toward the development of organization-wide infrastructure of parent and family engagement. We also outline the values and mechanisms that were perceived as the building blocks of the culture of engagement at CanChild. Finally, we summarize the top ten issues encountered in the course of working together, and some examples from our research studies of how these issues could be addressed. In our Discussion we have selected a few important milestones and studies that exemplify key trends in our trajectory of parent–researcher relationships. These studies reflect the knowledge and experiences of the authors, since people were more likely to offer deeper and more nuanced reflections on the studies in which they themselves have been involved (as investigators, family partners, trainees or research staff).

### Evolving role of parents in research studies

The roots of family engagement at CanChild go back to the mid 1990s when parents first began to be involved in research studies. At the time, the tenets of involving patients and families in health research were already being formulated in the UK, but not yet in Canada. For example, the UK’s National Institute for Health Research established a national advisory group on this topic in 1996, while in Canada, the first formal attempt to articulate a Strategy for Patient-Oriented Research (SPOR) took place over a decade later, in 2011. Therefore, at CanChild, the desire to involve families was not driven by the top-down impetus towards patient and family involvement in research. Rather, it was inspired by the clinical focus in children’s rehabilitation that was then starting to include the entire family. At the time, a group of CanChild researchers were developing a definition and a measure of family-centred service, the core tenets of which recognize the expertise of parents in their child’s health and needs, and the importance of focusing on the family context [25]. To the researchers leading this study, it logically followed that since families were recognized as valuable partners in the delivery of health services to their children, they should be seen as equally valuable partners in research [26]. In order to recruit the parents in this study, CanChild researchers connected with two provincial networks and three local children’s rehabilitation centres. Parents from these networks participated in both surveys and focus groups to share their experiences with the delivery of healthcare services, and a small group of them reviewed and provided feedback on the proposed tool. Three of these parents subsequently worked with researchers to co-produce a series of knowledge translation tools on family-centred service [27]. These tools are housed on the CanChild website and are used in training workshops for service providers.

In the early 2000s, parents began to be more fully engaged throughout the different phases of research studies and to assume more decision-making roles. A good example of this process is offered through two related research studies, Move and Play (2006–2009) [28] and On Track (2012–2017) [29]. The Move and Play study followed children with cerebral palsy in Canada and the United States to gain an understanding of factors associated with motor function, participation and play. At the time, two parents reviewed and provided feedback on grants, training and recruitment materials, and knowledge products including presentations and research summaries. After the first study’s completion, the research team began to plan the follow-up On Track study, which aimed to capture and describe developmental trajectories among children with cerebral palsy. With the planning of the On Track study, the team had evolved in their thinking about the role of parents, both as a result of their previous experience of working together, as well as informed by the newly emerging literature and guidelines around patient engagement in research. For example, a research grant from the Patient-Centred Outcomes Research Institute (PCORI), funded in 2010, both enabled and mandated the creation of a parent advisory committee composed of seven parents (including two parents from the previous Move and Play study and five additional parents). Although the research questions were still generated by the researcher/clinicians (based on existing gaps in the literature), the parents’ experiences and priorities informed the progress of the study and gave a new direction to the study’s knowledge translation and dissemination activities. For example, the parents wanted to ensure that a focus on motor developmental curves did not detract attention from the children’s future potential and strengths in other areas of life. The parents initiated and developed a series of tip sheets, videos, blogs and articles in an online parenting magazine in order to contextualize the study findings and highlight key take-aways from a parent perspective [29].

In 2011 CanChild embarked on another ongoing study where parent engagement evolved organically and was significantly driven by the parents’ own interest. The study began when two CanChild scientists co-authored an article to bring to life the World Health Organization’s framework for health, the International Classification of Functioning, Disability and Health [30]. Built on the concept of the “F-words in Childhood Disability”, the article features six key words (Function, Family, Fitness, Fun, Friends, and Future) that the authors state should constitute the focus in childhood disability. Following the article’s publication, one parent learned about the F-words concept through her online network, developed her own F-word tool and contacted CanChild directly to share it; another parent wrote a blog post about the F-words and was subsequently contacted by a CanChild researcher and invited to join the research team; a third parent was told about the F-words in clinic by a CanChild clinician researcher and joined the research team after expressing interest in it. As a result of these parents’ self-identified interest in the F-words, CanChild researchers partnered with them to develop, disseminate and evaluate a series of Knowledge Translation (KT) projects focused on the F-words [31]. Over time the F-words Knowledge Translation & Research Program has grown and there are now many parents who have contributed and partnered on F-words related research and knowledge translation activities, including: conference presentations; webinars; educational outreach visits/online training; and a current research study being conducted in partnership with five organizations to evaluate the implementation strategies and overall impact of the F-words on families and service providers [32].

In the past five years, more research studies began to engage parents in planning and decision-making roles. In 2018, CanChild partnered with Kids Brain Health Network (KBHN) and McMaster Centre for Continuing Education to develop a training course in Family Engagement in Research [33]. The course content was co-developed by a researcher and two parents, all of whom are also named as co-investigators on the study, and are currently delivering the course together. This model of researcher-family partnership is also applied in other recent studies that involve parents as co-investigators from the planning and grant-writing stages. One of these studies aims to develop a measure of family-centred service; the other aims to adapt an online early intervention program for parents (ENVISAGE) for service providers (ENVISAGE-SP), a program that current parent participants identified as a necessary next step [34]. Both research teams have a parent co-principal investigator along with several parent co-investigators.

The examples cited above show that over time, the roles and responsibilities of parents have evolved from being consulted on research studies to undertaking decision-making roles as partners and recently as co-investigators. This evolution was enabled and supported by new research and best practices in patient and family engagement. For example, the Canadian Institutes of Health Research (Canada’s federal funding agency) has recently started to require that studies involving knowledge users (including patient and family partners) must identify at least one Principal Applicant who is a knowledge user [35]. However, this does not mean that all patient and family partners must commit themselves to undertaking such roles; on the contrary, recent guidelines and frameworks in patient and family engagement emphasize the necessity of choice and flexibility in roles and responsibilities [36].

### Beyond the research study

Over the last decade CanChild has started to develop an infrastructure of parent engagement that goes beyond the individual research study. For example, CanChild’s Advisory Board has a parent (as well as a youth) representative on it, and CanChild-affiliated families (including parents and youth) were invited to the organization’s most recent strategic planning retreat in 2019. Several CanChild staff members are also parents of children with disabilities and bring their lived experience into their work.

A particularly noteworthy initiative that allows for ongoing connections and interactions between families and researchers was the creation of a private Facebook group, at the time called ‘Parents Participating in Research’, a name later changed to ‘Parents Partnering in Research” (a change which in itself reflects the evolving perception of the role that parents play in research) [37]. Launched in 2014, the group was originally envisioned as an advisory group to CanChild that would facilitate active engagement from family members and knowledge exchange regarding “project planning, research direction, the current state of special needs parenting, supports, and services, as well as how to translate research knowledge to best serve parents and youth living with disability” [20]. The parent who spearheaded the group reflected on the group’s multiple purposes in these words:

[T]here's got to be a way that we could do this, that not only could we bring a whole bunch of different parents together whose kids have different diagnoses, but that have similar experience…. And we could help them understand the research process, and maybe engage them a little bit longer…. I was hoping that it gives people who didn't think that they had a voice prior to this, the opportunity to speak. I'm hoping that it brings in a lot of different perspectives on childhood disability from across the country…. And I'm hoping that it really empowers people to bring lived experience into it…. I'm hoping it's going to give people a voice and a place to talk about things and to get ideas generated. Lots of times I was looking for research papers on all sorts of different things. But they weren't there. You know, is this a topic that somebody could be looking into that might be a good place to start. [Parent]

The group is moderated by a parent and at the time of writing has 270 members, the majority of whom are parents. However, a number of researchers from inside and outside of CanChild also participate in the group, as well as individuals who “wear multiple hats” as both parents and researchers or research trainees. Over time, the group has evolved to become much more than an advisory group. Members share information about new research initiatives (studies, events, conferences, publications), discuss news and issues in childhood disability, ask questions, and sometimes simply share experiences. We see this diversity of functions as indicative of an organic community of engagement where parents and researchers can build relationships and learn from each other. One researcher, for example, reflected that participating in the Facebook group opened his eyes to issues that he does not hear in clinic, since the topics discussed are driven by the interests and concerns of families rather than researchers or clinicians.

[I]n that Facebook group I really had this aha moment that I'm hearing stories of families, that in my career of 20, 30, I don't know how many years, I haven’t heard in clinic…. What are the actual struggles? And these might be minor things. Somebody’s washing machine is broken and laundry piled up and husband is away because he works far away and she has these two kids, one is autistic… and all these little things that parents never talk about when they come to clinic… I felt privileged in being able to listen to these stories because I learned from that. And I cannot learn this in my clinic, although I’ve been seeing so many patients over so many years. [Researcher]

Upon reading a draft of this article, the original author of the aforementioned “laundry comment” reflected in an e-mail: “[It’s] funny to think my post about a pile of laundry and a husband away actually influenced someone to change the way they do things…. It shows that what we tried to do actually did make a difference.”

Since the launch of the PPR Facebook group, several other CanChild research studies have experimented with starting their own Facebook groups to allow participants to exchange ideas outside of study meetings. However their long-term future beyond the research study is uncertain, since they do not get a lot of traffic and require additional staffing resources. This suggests that rather than creating multiple siloed groups, perhaps we should be thinking about creating and supporting a larger community of engagement composed of patients, families and researchers from different organizations.

### Beyond the organization

The next frontier in forging a culture of patient and family engagement is creating an infrastructure of engagement that transcends not only the individual research study, but also the individual organization. As more and more health research is carried out through collaborations between institutions and large national and international networks, these institutions are also starting to engage patients and families on a network level. In the Canadian childhood disability landscape, such collaborations are creating new opportunities for partnerships and knowledge exchange among patients, families and researchers. For example, since 2012, CanChild has co-ordinated a Stakeholder Advisory Group composed of patients and family partners for a provincial cerebral palsy research network CP-NET [38]. The group advises on research activities that happen at the network level, bringing together researchers and family partners working on studies related to cerebral palsy. Some of its activities include helping to organize the network’s annual Science and Family Days and other knowledge mobilization initiatives such as webinars.

Another initiative that seeks to bring together families and researchers interested in childhood disability is the aforementioned Family Engagement in Research (FER) training program. The cornerstone of the program is an online course that trains researchers and family members in the principles of patient and family engagement. At the time of writing, 163 participants from nine countries have completed the course. Many graduates from the course continue to keep in touch through a monthly online newsletter [39], and several parent graduates have since become engaged in new research initiatives stemming from the connections they have made in the course. The course format is currently being adapted by a Dutch research team affiliated with CanChild for use in the Netherlands, and other adaptations are being considered for other national/international organizations.

The FER training program is now expanding to include additional knowledge mobilization initiatives such as a monthly research and discussion forum that aims to deliver relevant research directly to patients and families. The forum, titled Luke’s Legacy Family Research Rounds [40], is led and moderated by a parent who hosts a different research team each month to present on topics and issues relevant to patients’ and families’ interests and concerns.

Taken together, the trajectory of studies outlined above illustrates the shift in thinking about parents as “advisors” or “collaborators” on individual research studies to starting to build a culture of engagement with parent partners not only on individual research studies but also the level of the organization and beyond. We now turn to examine the building blocks of this process: the values that underpin our work and the mechanisms through which these values are entrenched and perpetuated.

### A culture of engagement

The overarching theme that emerged in the interviews is the presence of certain values that are seen as central to the work of CanChild researchers. These values include an openness to learning from others, a commitment to relationship-building, and the drive to “do better”. CanChild researchers and staff repeatedly talked about wanting to learn about, and from, parents’ experiences, and underscored the value of what parents bring to the table [42]. As one research staff member put it:

When you work with [Researcher X] you just get that everybody brings something. No matter if it’s [a] high school student or a parent, or someone who is world renowned in their field. So I think there's a lot of philosophy of that around here, people are always trying to figure out what other people can contribute. [Staff member]

This openness can take the form of both “seeking” and “embracing”; that is, researchers are on the lookout for parents who might bring something new and valuable to childhood disability research, and they also embrace and invite on board parents who bring forth their ideas or interest. Of the eleven parents involved in writing this article, four found their way to CanChild through a CanChild researcher: two at conferences, one in clinic, and one in a social setting with her child. Increasingly, the parents themselves contact CanChild to express an interest in getting involved in research. When that happens, being able to offer parents a variety of opportunities that are not limited to one research study (for example a Facebook group, a newsletter, or a research forum) is a good way for interested parents to learn more about childhood disability research in general and CanChild in particular, to connect with other parents and researchers, and to learn more about current research studies and other initiatives.

Another key value that emerged as the cornerstone of a successful parent–researcher experience is the commitment to building relationships. While that sounds like a truism, building and maintaining relationships takes time and work. One parent described her relationship trajectory with a graduate student in these terms:

We were learning, both of us were learning what to do. She didn't know how to behave with parents, it was completely new. I didn't know what she's expecting from me…. She was a natural. She never made me feel like I am doing something wrong or I'm late, it was on my pace…. Me and [Researcher] we built that relationship to a beautiful one. So I was not scared to share something…. Because when you're open and talk about stuff and just throw ideas on the table, one of them will be great. Few will not. So that the relationship with the lead investigator is very important. They need to know how to empower parents, how to talk to them, and make them feel that they're not used.

Although time is a chronically scarce resource in academia, taking the time to connect with parent partners as “people first” [43] goes a long way towards a successful relationship. In fact a number of parents identified their friendship with the researchers as the primary factor that smoothed over many of the wrinkles that often crop up in the course of a research study.

Researchers were reflective about the needs and priorities of the families with whom they work and regularly expressed the desire to learn and “do better”. Parents’ insights sit with them and shape their thinking about both present and future research studies. One research coordinator reflected on the trajectory of parent engagement on her study:

We know we're not doing the best that has ever been done, we know that we can always do better, but tell us how, let's learn together and let's try to do better, even if we're not going to do the best. Let's not just accept that it is what it is, or we can only afford to do this much so we just won’t do that… We want it to be reciprocal and we want it to be a good experience, and we want to improve the way that we’re doing things. And that’s what keeps people around I think, knowing that you can be humble and say maybe we could have done that better, or maybe we have a lot to learn still, but let's try and figure it out together. [Research coordinator]

The above comment captures well the sentiment articulated by many researchers. They are committed to working with parents, aware of the limitations and shortcomings to “ideal” engagement, yet driven to learn and do better. One trainee who has worked with parents in various studies for over a decade reflected on her own learning trajectory:

I've learned so much more now that I could do such a better job now than I did back then… I know a lot more about the science and the strategies to put into place, whereas at the time I didn't have any training in how to engage parents as partners on the project.

The values of openness to learning, relationship-building and a commitment to doing better, emerged in the interviews as the pillars of CanChild’s work with parents. These values are espoused by the leadership, instilled in the next generation of researchers, and inform both research and clinical work. Researchers, staff, trainees and parents alike credited CanChild’s past and current leadership for fostering the type of organizational culture where everyone’s views and contributions are valued. One parent with extensive research experience noted the importance of multi-directional effort in order for the principles of patient and family engagement to truly inform research.

You need both bottom up, you need the trainees to push, but you also need buy in from the top... But it does come from the philosophy from whoever was at the top.

The trainees with whom KP spoke underscored that they were socialized by their mentors into a particular way of interacting with parents—values that they wanted to carry forward in their own doctoral projects and beyond, even when it added considerable time, work and complexity [44]

[Researchers X and Y], they're so supportive. You know, it's all about trust and communication and transparency, like these values that they ingrained in me from the very beginning that were going to be important [Trainee]

Another theme that we noted is the way in which research and clinical work inform each other. Clinicians talked about learning from parents in clinic and having parents’ experiences inform their research questions and priorities. One staff member reflected:

It's probably the leadership that we have that just thinks that way in terms of their clinical work…. Like for [Researcher X] for instance, that's just part of the way that he does things, in the way that he speaks directly to children when he sees them in his clinic and involves the parent from the beginning. So it's just something that was a no brainer for him that if you're doing research about families that of course you need to have input from families. [Staff member]

Above we have set out the values that drive researchers’ work with parents, and the ways in which these values are put into practice. At CanChild, parent engagement has evolved organically (and somewhat serendipitously), based on the intrinsic motivation of researchers and parents. Our study has revealed the presence of certain “soft” building blocks—such as a culture of openness, or a commitment to relationship-building—that cannot be mandated by funding bodies. However it must be acknowledged that these “soft” values were able to take hold because they were espoused by CanChild leadership, who in turn set the tone for a particular organizational culture in which subsequent generations of researchers were trained. Furthermore, the development of guidelines and strategies (most notably, the Canadian Institute for Health Research’s SPOR strategy in 2010 [41]) has been helpful to foster practices that were not possible in health research a few decades ago, such as including salaries for patient and family partners as co-investigators on research grants.

As our trajectory of working together illustrates, parent engagement is a work in progress. Below we identify the key issues that parents and researchers identified as important to consider when working together, and share some possible strategies and facilitators for working through them.

### Key issues and possible strategies and facilitators for working through them

In what follows, we outline the “top 10” issues (in no implied order of importance) that we have encountered in the course of working together on research studies. Many of these themes are echoed in the existing literature [1, 12, 45–48], a fact which illustrates the ubiquity of these issues and the continuing need to address them in the work we do.

#### Lack of familiarity with research culture

A number of parents on our team noted that the lack of familiarity with how things work in the research world makes it difficult for parents to understand what is expected of them and to feel like they are part of the team [47]. Most parents, even though they may be highly accomplished professionals in fields such as business or law, have never encountered the intricacies of ethics reviews or academic publishing. One possible strategy for introducing or on-boarding parents to the research world is to assign a mentor for parents who are new to research. On research teams consisting of more than one parent, a more seasoned parent can serve as a mentor to the newcomer parent. Organizations can also consider having a designated parent whose role is to mentor and on-board parents. At CanChild, the Research Engagement Strategist (a parent herself) is an informal mentor to parents who want to explore possibilities in research or who have questions or concerns. Over the past three years, participants in the Family Engagement in Research course have also developed a number of tools which can be useful for introducing parents to the research process and lingo [49].

#### Parents’ lives are complex and unpredictable—and every parent is different

Parents who commit to taking part in research studies take their commitments very seriously; in fact, they might be reluctant to commit in the first place for fear of not being able to follow through due to their caregiving obligations. Sometimes parents need to take time out of research for personal or family reasons. Every parent is different: everyone has a different story, professional background, needs, and expectations [47]. Thus, researchers who work with parents need to build considerable flexibility into project tasks and timelines (for example, by providing accommodations that help facilitate parents’ availability by offering to meet in the evenings or on weekends, or by offering asynchronous opportunities for engagement). Researchers leading the study need to create an atmosphere where everyone is comfortable discussing roles and expectations, and these conversations need to continue throughout the study to allow for parents’ changing situations and life circumstances. As noted above, many tools exist that can be used to facilitate these conversations: one CanChild favourite is the Involvement Matrix [50], developed by a Dutch parent–researcher team affiliated with CanChild. The Matrix illustrates the different ways in which patient/family partners can be involved at various stages of the research, on a continuum from “listening” to “making decisions”. A terms of reference document can be used to outline roles and expectations, as well as assure patient and family partners of the possibility to decline activities and to take a leave from research responsibilities as needed.

#### Power imbalance

The researcher–parent relationship is characterized by an inherent power imbalance [12, 47, 48]. Even highly-educated parents with professional backgrounds disclosed having at times felt insecure, intimidated and that “they’re just a parent”. Interestingly, a few among us noted the existence of analogous “just a clinician” or “just a trainee” feelings among researchers. While these latter sentiments do not obliterate the inherent power differences on a research team, they do suggest that taking time to build relationships, to know each other as “people first”[43], and to acknowledge explicitly each other’s contributions, can help even out the playing field [51, 52]. A number of tools exist to help attune researchers to the factors that contribute to power imbalances, and some strategies to mitigate them [53, 54].

#### Relevance of research

Several parents noted that research questions do not always reflect parents’ realities and priorities. At times parents’ feedback gets incorporated, whereas at other times it does not, with little explanation as to why. One parent emphasized that researchers have a responsibility to be activists and to help speak out on issues that are important to parents.

Researchers, for their part, truly do want to produce research that is meaningful to patients and families and for these individuals to benefit from the process. One example of a study that arose in response to a gap identified by participants was MyVoice, a qualitative study that explored the lived experience of young people with cerebral palsy. The study was initiated because participants involved in a previous study that sought to understand the implications of having cerebral palsy on participation, brain function and development, felt that their experiences were not adequately captured through the quantitative methods employed [55].

Researchers are also concerned about making sure that study results are meaningful and relevant to patients and families. One research coordinator reflected that she tries to “give back” to parents by disseminating relevant information through social media, rather than assuming that parents will obtain this information from journal articles. One trainee suggested that one way to make research more relevant would be for funding organizations and institutions to require research studies to produce knowledge translation products aimed at the broader community. The aforementioned example of the OnTrack study, in which parents steered dissemination and knowledge translation activities in the direction relevant to them, illustrates how researchers can make a conscious effort to support the creation and dissemination of research that patients and families actually want and need.

#### Tokenism

Both researchers and parents were attuned to the dangers of tokenism [56]. Researchers, for example, were concerned that top-down requirements from funding organizations may reinforce “just checking off the boxes”, particularly in the case of large multi-site studies where deadlines, distance and bureaucracies can impede in-depth conversations and relationship-building. Several researchers and staff suggested more training in family engagement for all parties involved in a research study, from principal investigators to research assistants to knowledge users. In all, we recommend that funding bodies, institutions and researchers need to budget the time and resources for relationship-building, engaging stakeholders from the planning stages, and providing training as needed.

#### Representativeness

It is well documented that the majority of parents who partner in research studies hail from relatively privileged demographics in terms of education, socioeconomic status, geographical location (with less representation from remote and rural areas), and ethnic background [47, 57, 58]. In childhood disability research, the majority of family partners also tend to be mothers. Much work remains to be done to engage parents from underrepresented populations, including minority groups and vulnerable populations. While we do not see any quick or easy solutions to this, as an organization we have recently started reaching out actively to organizations that work with populations who are under-represented in research. Through these initial conversations, we aim to build relationships and learn about each other’s strengths, concerns and needs. Another noteworthy initiative at McMaster University is the development of a Co-Design Hub for vulnerable populations [59]. The hub, spearheaded by a number of CanChild researchers, aims to facilitate partnership formation, advance methods of co-design, and enable knowledge-sharing with various vulnerable populations.

A related issue in the engagement literature is that the same patient and family partners tend to be involved in multiple research studies [60]. How do researchers ensure a variety of voices and perspectives and avoid over-burdening those who are part of their circle? At CanChild, the FER course in fact created the opposite challenge: a fresh supply of trained and interested parent partners who do not know where to find research studies. Some organizations and research networks have developed databases or “matching services” that allow interested family partners and researchers to connect [61]. We see the value in these initiatives and ponder how they could be grown on a larger scale without compromising the organic relationship-building that we see as the key to successful partnerships.

#### Dissonance between rules and requirements at different levels

Universities, funding organizations, and other study partners have their own rules and requirements that are not always in accordance with each other, and may not be conducive to implementing the principles of family engagement. They, too, are always in the process of evolving, so that these mandates and requirements are also a “work in progress”. For example, a funding organization may mandate compensation for family partners but a university’s payroll structure may not be set up to allow for such roles. While researchers cannot single-handedly solve all of these issues, those with greater institutional clout can use it to chip away at bureaucratic hurdles as much as possible to advocate within their own spheres of influence. Researchers should also openly and honestly communicate with patient and family partners about the “behind the scenes” procedures and intricacies that may complicate the engagement process.

#### Time

Communicating with parents, preparing information for meetings, obtaining and implementing parents’ input, “checking in”—all take time. Parent engagement is not a linear process and may entail going back a few steps, for example if a parent misses a meeting and needs to be brought up to speed. While the researchers in this study believe that working with parents is worth the time and effort, they do suggest that extra time for meaningful engagement, including staff resources, needs to be built into project timelines and funding models.

#### Compensation

Financial compensation offers recognition and validation for one’s work. Numerous barriers to compensation exist in health research, including: lack of funding outside of research studies (including in the grant preparation phase); lack of recognition of the patient/family partner category by human resources departments at research institutions; and the sometimes still-prevailing sentiment that receiving compensation for one’s work is antithetical to “authentic” desire to partake in research [60]. However, while the parents in this study emphasized that money is not their primary motivation for engaging in research, they also noted that not being compensated can make one feel “less than” [62]. In recent years, organizations such as PCORI in the United States, the CHILD-BRIGHT Network in Canada, or INVOLVE in the United Kingdom have developed guidelines for compensating knowledge users for their involvement, and these have been adopted in various CanChild studies.

#### An infrastructure for engagement

An increasingly prevalent theme in the literature on engagement is the importance of patients and families themselves informing the research questions and the planning of the studies. Incorporating partners from the planning stages helps to foster authentic partnerships and reduces tokenism. But how are researchers to find parents and compensate them for their time even before the study is funded? Inevitably, having parents on board from the planning stages requires a pre-existing community of engagement as well as financial resources that transcend individual study grants. At CanChild, patient and family representatives sit on the Advisory Committee and were involved in developing the organization’s most recent strategic plan. Furthermore, initiatives such as the FER program and the PPR Facebook group provide opportunities for developing long-term relationships with families.

Taken together, the insights and reflections of parents, researchers, staff and trainees reveal that parent engagement is both about *being* (that is, holding a particular set of values) and *doing* (that is, putting those values into practice). Ideally, the latter should flow from the former, and it often does. However, deadlines and bureaucracies can get in the way, especially in case of large multi-sited studies involving multiple institutions and funding bodies. Themes that weave through every single issue identified above include the importance of open and honest communication and relationship-building, and the need for infrastructure to implement this [63]. Researchers who want to work successfully with parents need to invest the time and energy into those relationships, which in turn requires financial and institutional backing. Thus, the facilitators to doing engagement are both relational and structural [64].

#### Considerations and next steps

An important consideration is that our study inevitably reflects the voices of those researchers, staff and parents who have considerable experience and success with working together. We can anticipate that researchers who do not engage parents in their studies, or who may not have had positive experiences with parent–researcher partnerships, may not have volunteered to be involved in this study. In a similar vein, the parents among us who agreed to participate are those who have had mostly positive experiences with CanChild studies/initiatives and thus chose to remain connected with CanChild. While we cannot know whether anyone has chosen not to participate due to their negative experiences with parent–researcher partnerships, we made an effort to be candid about the challenges we faced, with the expectations that others whose voices are not reflected in this article may have experienced some of the same issues.

Another consideration to address is our decision to focus this article on CanChild’s trajectory of engaging parents, as opposed to other groups (patients, other family partners and service providers). Admittedly, CanChild has a much longer track record of working with parents than patients (in our case, children and youth) or other family partners (e.g., siblings or grandparents). However, several initiatives are currently underway that will expand our focus beyond the parent population. For example, CanChild’s Advisory Board has a youth member on it, and the current ongoing CHILD-BRIGHT READYorNot^TM^ Brain-Based Disabilities Trial (which developed, and is now evaluating, an app to for youth transitioning from pediatric to adult healthcare) has a patient and family advisory committee that includes eight youth members [65]. Furthermore, CanChild researchers are currently working with a group of youth to develop training materials on patient and family engagement for the youth population, and a PhD student is working with a committee of sibling partners on a study focusing on the experiences of siblings of youth with disabilities during the transition to adulthood [66].

Finally, we recognize that our use exclusively of qualitative methodology is still unusual for healthcare audiences. We believe that the methods used, and the relatively small sample of respondents, allowed us to capture nuance and depth that we might not have been able to capture quantitively. That being said, we are aware that different methods generate different types of knowledge. One of the strengths of qualitative research is that it identifies issues that can subsequently be probed quantitively if desired. Having learned a lot from doing this study, we are now planning another project that will examine our organization’s engagement strategy with all relevant stakeholders—not only parents, as has been done in this article—using a combination of qualitative and quantitative methods.

## Conclusion: a culture shift

Family engagement in research is part and parcel of a larger paradigm shift (or at least expansion) taking place in healthcare towards family-centred and patient-centred care, and in research towards more participatory approaches. In this study we set out to examine the trajectory of parent engagement at CanChild in order to learn how engagement is embedded in our organization’s infrastructure and what are the long-term effects of engaging parents on the culture of research. Our findings showed that CanChild’s work with parents is underpinned by the values of openness and a commitment to learning and improving. These values are embraced by the leadership, mentored and instilled into the next generation of researchers and permeate all levels of the organization, including both research and clinical work. However, while the values have been present from the start, the ways in which parents have been involved have evolved over time, reflecting the changing priorities and possibilities in health research.

Like all cultures, the culture of healthcare and health research is always in the process of change. Research institutions and funding bodies now formally recognize the role that patients and families play in research—for instance, by allowing family partners to be named as co-investigators on research grants or to co-present with researchers at conferences. Researchers are paying more explicit attention to how engagement is being done and are starting to see it as an integral part of healthcare research. The case study of CanChild suggests that cultivating a culture of engagement requires support and buy-in at multiple levels. “Soft” values such as openness and a commitment to learning and growing cannot be mandated by funding bodies, but they do need to be cultivated by those in leadership roles and instilled in the next generation of researchers. On the other hand, funding organizations can provide resources to enable an infrastructure conducive to true engagement. Furthermore, developing mechanisms for connecting researchers and patient/family partners beyond individual research studies helps to build a culture in which research can be truly informed by, and relevant to, the people about whom the research is being done.

## Data Availability

The datasets generated and/or analysed during the current study are not publicly available as they could compromise the confidentiality of individuals involved in the research studies discussed in the article.

## Appendix: Interview Questions

### Interview Questions for Researchers

1. How did you first start working with parents in research studies?
2. Can you tell me about the projects in which you’ve taken part that also engaged parents?
  Probes:
  - What was this project about?
  - In what capacity did this project engage parents?
  - In your opinion, have the insights of parent partners impacted the decisions of the project team?
  - In what area(s) do you think parent involvement contributed the most?
  - Are there any new things that you learned from the experience of partnering with parents on this project?
  - What surprised or challenged you with regards to engaging parents in this study?
3. What does it mean to you for parents to be engaged as “partners”? Do you think a true partnership is possible? If yes, what does it look like?
4. What insights did you gain from the experience of engaging parents that you would like to apply in future research projects?
5. What do you think of the way that CanChild engages parents?
  Probes:
  - When it comes to parent engagement, what do you think CanChild is doing well?
    What areas do you think need to be improved?
6. How has parent engagement in research evolved since you started carrying out research?
7. Where do you see parent engagement heading in the future?
8. What effect do you think parent engagement has on research projects?
9. What effect do you think parent engagement has on research institutions?
10. What effect do you think parent engagement has on the culture of research more broadly?
  Probes:
  - Engaging parents in research is often advocated on the grounds that it makes research more relevant and democratic. What do you think of that idea?

### Interview questions for parents

1. How did you first get involved in research?
2. Can you tell me about the project(s) that you’ve been part of?
  Probes:
  - What was the project about?
  - What was your role in this project?
  - Did you learn any new things from the experience of taking part in this project?
  - Did you encounter any surprises or challenges?
  - Did you feel that the team listened to and absorbed your input, and that your insights impacted the decisions of the team?
3. What advice would you give to other parents who are considering getting involved in research?
4. Prior to becoming a parent, were you in the paid workforce? What sort of work did you do?
5. Are you currently in the paid labour market?
6. Have you ever done any volunteer or community work? If yes, can you tell me more about that?
7. What skills/knowledge do you feel that you brought into the research projects that you’ve been part of?
8. Do you feel that participating in this project allowed you to develop any new skills/gain new knowledge? Can any aspects of your participation help you in your future personal or professional undertakings?
9. What has your experience with engaging with CanChild been like?
  Probes:
  - When it comes to parent engagement, what do you think CanChild is doing well?
    What areas do you think need to be improved?
10. Has your involvement in healthcare research changed your life in any way? Has this particular project changed you in any way?
  Probes:
  - Do you see yourself differently?
    Does it change the way you interact with service providers?
    Do you think that other people (for example, service providers) see you differently?
11. What do the people closest to you (spouse/significant other, children, parents) think about your involvement in research?
12. Do you think you will ever take part in any more research projects? Why or why not?
